# Structured viva validity, reliability, and acceptability as an assessment tool in health professions education: a systematic review and meta-analysis

**DOI:** 10.1186/s12909-023-04524-6

**Published:** 2023-07-25

**Authors:** Abdelhamid Ibrahim Hassan Abuzied, Wisal Omer Mohamed Nabag

**Affiliations:** 1 Faculty of medicine, University of Khartoum, Alqasr Ave, P.O. Box 102, Khartoum, Sudan; 2 Faculty of medicine, Alzaiem Alazhari University, Khartoum, Sudan

**Keywords:** Structured viva, Validity, Reliability, Acceptability, Health professions education

## Abstract

**Background:**

The viva, or traditional oral examination, is a process where the examiners ask questions and the candidate answers them. While traditional viva has many disadvantages, including subjectivity, low validity, and low reliability, it is advantageous for assessing knowledge, clinical reasoning, and self-confidence, which cannot be assessed by written tests. In order to overcome these disadvantages, structured viva was invented and is claimed to be highly valid, reliable, and acceptable, but this was not confirmed by an overall systematic review or meta-analysis of the studies. The research aims to investigate the studies to reach an overall decision regarding the quality of structured viva as an assessment tool according to the agreed standards in medical education in terms of validity, reliability, and acceptability.

**Methods:**

This systematic review was done following the Preferred Reporting Items for Systematic Reviews and Meta-analysis (PRISMA) guidelines. PubMed, Best Evidence Medical Education (BEME) website reviews, Google Scholars, and ScienceDirect databases were searched for any article addressing the research questions from inception to December 2022. Data analysis was done by the OpenMeta Analyst open-source app, version Windows 10.

**Results:**

A total of 1385 studies were identified. Of them, 24 were included in the review. Three of the reviewed studies showed higher validity of structured viva by a positive linear correlation coefficient compared with MCQs, MCQs and Objective Structured Clinical Examination (OSCE), and structured theory exam. In the reviewed studies, the reliability of structured viva was high by Cronbach alpha α = 0.80 and α = 0.75 in two different settings, while it was low α = 0.50 for the traditional viva. In the meta-analysis, structured viva was found to be acceptable by overall acceptability of (79.8%, P < 0.001) out of all learners who participated in structured viva as examinees at different levels in health professions education using the available numeric data of 12 studies. The heterogeneity of the data was high (I^2 = 93.506, P < 0.001) thus the analysis was done using the binary random-effects model.

**Conclusion:**

Structured viva or structured oral examination has high levels of validity, reliability, and acceptability as an assessment tool in health professions education compared to traditional viva.

## Introduction

Assessment of the students is a cornerstone in medical education science and thus proper.

assessment is crucial to get quality medical graduates who eventually meet society’s needs and promote the health of the community [1]. Traditional Viva, Viva-voce, or traditional oral.

examination is a process between the examiners who ask questions and a candidate who must.

reply to them [2].

Viva or oral examination is popular as it is a part of many undergraduate and postgraduate programs in health professions education. It is usually used in situations like the decision to pass or fail marginal students in basic sciences, giving a prize to the best student as well as in defending the theses.

The disadvantages of traditional viva include poor content validity, low inter-rater and inter-case reliability, inconsistency in marking, and lack of standardization. However, studies have shown that the validity and reliability can be increased by using structured standardized or structured formulae [2].

Structured viva was properly described as a separate assessment tool by Oakley and Hencken in 2005, but it was described and used in health professions education as early as 1993 by Thomas et al. and 1989 by Tutton et al. [3–5].

Structured viva has the advantage of being structured, objective and it is claimed to be fair and reliable, but this was not confirmed by an overall decision such as a systematic review or meta-analysis of the studies [6]. Generally, viva usually assesses knowledge (recall), in-depth clinical reasoning and attitude of the candidate on specific topics, and self-confidence which cannot be assessed by written exams [7]. It should not be influenced by age, gender, race, or socioeconomic status [8].

Reliability is the ratio of the true score variance to the observed score variance, it can be measured with Cronbach alpha which measures the internal consistency of marks of an assessment tool, a value with 0.8 or more considered reliable. Some educationists use the reliability coefficient to measure reliability [5].

Validity is whether the tool measures what is supposed to measure and the reliability is the ratio of the true score variance to the observed score variance [9].

According to the standards in medical education; validity, reliability and acceptability are considered parts of the criteria used for determining the usefulness of a particular method of assessment, together with the feasibility and educational impact those five elements are the criteria of good assessment tool [10].

All of the assessment methods have strengths and intrinsic flaws. Viva or oral examination is widely used in health professions education thus, the aim of this review was to provide a further summary and overview of the studies that have assessed structured viva as an assessment tool in health professions education in terms of validity, reliability and acceptability in comparison to other forms of assessments.

The conceptual framework of the study is to systematically review all the studies related to structured viva validity, reliability and acceptability and to do a meta-analysis following the PRISMA guidelines and protocols which are mainly;

- Studies selection criteria including the flow diagram.
- Study characteristics.
- Studies bias,
- Study limitations.
- Fund.

All the study details following the PRISMA guidelines are mentioned step by step in the following sections. The importance or purpose of studying this topic is to ensure the quality of structured viva as an assessment tool according to the agreed standards in medical education in terms of validity, reliability and acceptability thus ensuring learners’ competency, fairness and medical education development.

## Materials and methods

This review was conducted according to the Preferred Reporting Items for Systematic Reviews and Meta-Analyses (PRISMA) guidelines. The databases of PubMed, Google Scholar, Best Evidence Medical Education (BEME) website reviews, and ScienceDirect were used for the systemic search for any published article in English addressing the research question till December 2022. If there is a disagreement between authors about what research to include, the research is reviewed in detail against all inclusion and exclusion criteria step by step. There were no sources of fund.

The search formula was done using all The Keywords “Viva”, OR “Viva voce”, OR “Structured Viva”, OR “Structural Viva”, OR “Structured oral examination” AND “Validity” OR “reliability” OR “acceptability” AND “Medical” OR “Nursing” OR “Dental” OR “Pharmacy” OR “laboratory sciences”. Words “OR” and “AND” were used as they are functioning in the database algorithms. Overall, all research of structured viva or structured oral examination in any health profession addressing the validity, reliability, and acceptability were searched.

### Inclusion criteria

All studies published in English in which structured viva was evaluated for validity, reliability, or acceptability of structured viva as an assessment tool in health professions education were included.

### Exclusion criteria

1. Traditional viva exams.
2. Online/Virtual structured viva exams.
3. Semi-structured viva exams.
4. No available abstract or full theses.
5. Articles not in the English language.

All articles retrieved from the search were screened for inclusion in this review based on their titles and abstracts. After that, relevant studies were reviewed for inclusion (full text) according to the eligibility criteria. The researchers assessed the quality of the included studies using the Medical Education Research Study Quality Instrument (MERSQI), a validated study tool used to appraise the methodological quality of medical education studies based on ten items reflecting six domains: study design, sampling, data type, the validity of the evaluation instrument, data analysis, and outcomes.

The researchers screened the articles by themselves and the two researchers participated in quality assessment blindly.

We aimed to assess the validity, reliability and acceptability of the structured viva. Therefore, we summarized the data of correlation coefficients, Cronbach alpha values, and percentages of learners’ acceptability of structured viva in the reviewed studies. The pooled summary prevalence was calculated from the random-effects model due to the notable heterogeneity. The statistical analysis was carried out using OpenMeta[Analyst] app Windows 10 version which is a completely open-source, cross-platform software for advanced meta-analysis.

## Results

A total of 1385 studies were found. Relevant titles and abstracts were screened following the inclusion and exclusion criteria and resulted in 60 relevant articles. After reviewing the screened 60 articles, 24 were found to meet the inclusion criteria (1, 5–6, 11–31).

**Fig. 1:**
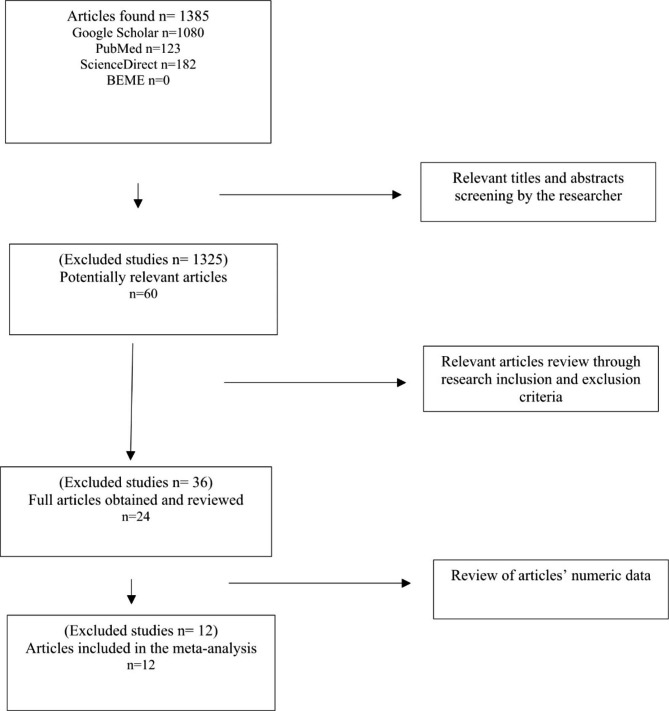
Flow of study selection and analysis through different phases of systematic review and meta-analysis

The mean MERSQI score for the 24 studies was 12.16 out of an 18-point scale. The schematic flow of the studies selection process is presented in Fig. 1 and the main characteristics of the 24 articles included in the review are in Table 1.

**Table 1:** Descriptive summary of the studies included in the review

Author	Year	Sample size	Students’ college	Students level	MERSQI
Madhukumar al.	2022	130	Medicine	Year 3	12
Chhaiya et al.	2022	40	Medicine	Year 2	12
Khalid et al.	2022	92	Medicine	Year 1, Year 2	14.5
Ahsan et al.	2022	56	Medicine	Year 2	14.5
Mallick et al.	2020	91	Medicine	Year 1	11
Khakhkhar et al.	2019	135	Medicine	Year 2	10
Imran et al.	2019	135	Medicine	Year 2	14.5
Sadiqa et al.	2018	92	Medicine	Year 1, Year 2	12
Dhasmana et al.	2016	107	Medicine	Year 2	12
Ganji et al.	2016	135	Dentistry	Year 3, Year 4	12.5
Dangre-Mudey et al.	2016	50	Medicine	Year 2	11
Bhadre et al.	2016	50	Medicine	Year 1	12
Waseem et al.	2016	100	Medicine	Year 1	11
Bagga et al.	2016	74	Medicine	Year 2	11
Vankudri et al.	2016	26	Medicine	Year 3	12
Khilnani et al.	2015	123	Medicine	Undergraduate	13.5
Hashim et al.	2015	171	Medicine	Year 4	9.5
Sharad K Gor et al.	2014	120	Medicine	Year 2	8
Jefferies et al.	2011	68	Medicine	Postgraduate residents	14
Shenawi et al.	2013	100	Medicine	Year 1	13
Hye Rin Roh et al.	2009	54	Medicine	Year 3	14
Kearney et al.	2002	46	Medicine	Postgraduate residents	11.5
J Anastaki et al.	1991	23	Medicine	Postgraduate residents	15
Tutton et al.	1989	433	Medicine	Year 3, Year 6	11.5

### Validity

Three studies (22, 25–26) estimated the validity of structure viva by the correlation coefficient (the linear relation to a criterion variable). All of them showed a highly significant correlation coefficient (r = 0.52, p < 0.01) between the results of structured viva and MCQs. Another study showed a statistically significant correlation (0.48 to 0.51) with multiple choice questions MCQs and OSCE (27). A positive highly significant correlation (r = 0.442, p = 0.001) was seen between marks scored in structured viva and structured theory exam while it was not significant (r = 0.202, p = 0.151) for the marks of traditional viva and structured theory exam [28].

### Reliability

The reliability of structured viva in the reviewed health professions studies was determined by Cronbach alpha and the correlation coefficient. Cronbach alpha of structured viva was α = 0.80 compared to α = 0.50 to the conventional viva as described by Madhukumar et al. [1], it was α = 0.75 as described by Anastaki et al. (27). It reached as high α = 0.79 for parts of Jefferies et al.’s structured oral exam of postgraduate training. The reliability coefficient is (0.7 to 0.8) for the use of a structured rating procedure for viva compared to 0.3 to 0.4 for an unstructured viva exam while the multiple-choice test was usually (> 0.8) as described by Tutton et al. [5]. A study showed significant inter-rater reliability for each pair of examiners and each question (r = 0.78 to 0.91; p < 0.0001) (27). Another study showed Interrater agreement of 86.7% as described by Roh et al. [22].

### Acceptability

**Fig. 2:**
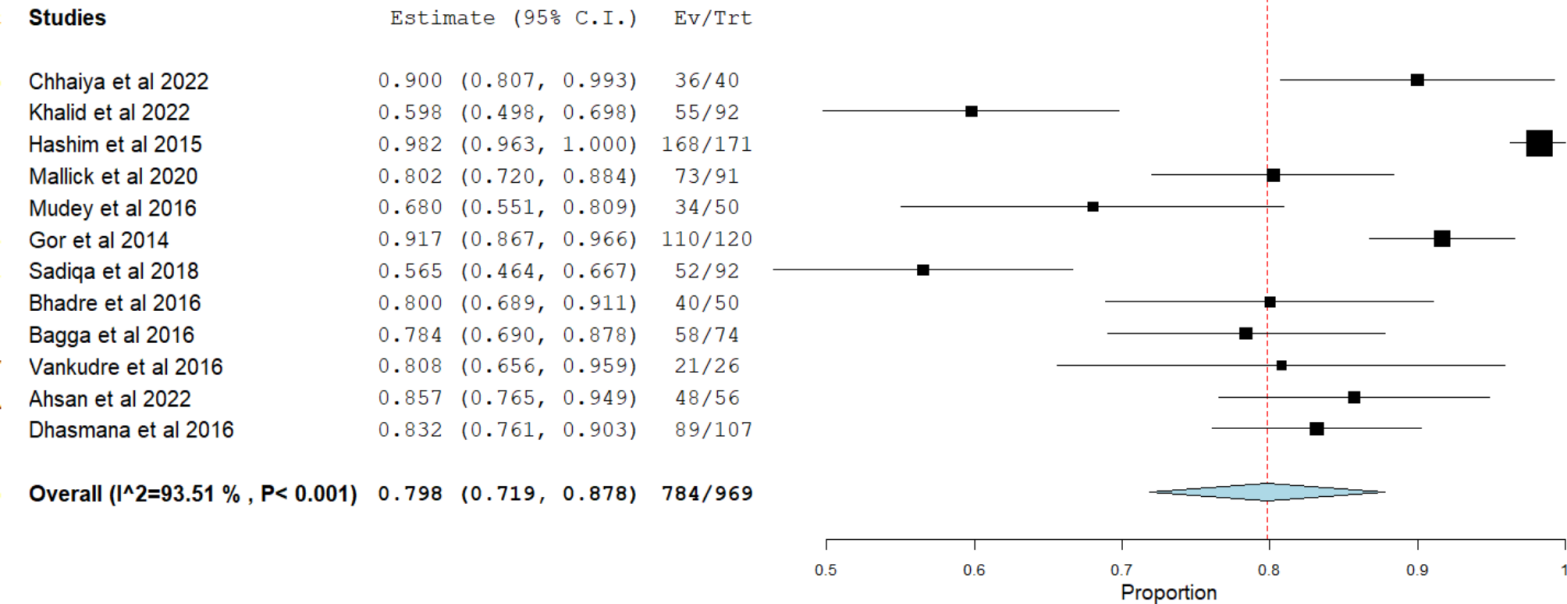
Forest plot analysis of the acceptability of learners of structured viva as an assessment tool

Twelve studies had sufficient data to calculate the overall acceptability rate of structured viva among the participants. The assessment tool was described as clear and fair and had a reasonable level of difficulty. Based on the available numeric data of the 12 studies, overall acceptability was (79.8%, P < 0.001) out of all learners who participated in structured viva as examinees at different levels in health professions education. The heterogeneity of the data was high (I^2 = 93.506, P < 0.001) thus the analysis was done using a binary random-effects model (Fig. 2). Begg and Mazumdar rank correlation Test also showed (Kendall’s Tau = 0.47) with.

p-value of (0.016), this indicates heterogeneity in the meta-analysis studies regarding the structured viva.

## Discussion

Health professions education requires learners to develop competencies in addition to knowledge such as professionalism, psychomotor skills, and communication skills. Therefore, the viva-voce examination is appealing because it gives the examiner an opportunity to assess a student’s depth of understanding and their ability to express it in a defined manner [2]. While education is the purposeful activities directed at achieving certain aims, assessment in education or health professions education is mandatory to ensure these aims for the quality and adherence to the standards for both individual learners and faculty members in order to improve performance through identification of areas for improvement and judging the individual competence. Students may be able to tolerate bad teaching, but they cannot tolerate bad assessment, and Assessment drives learning, so assessment in health professions education lies together with the curriculum learning objectives and learning methods as vital cornerstones of any curriculum [32]. One of the causes of differences in the assessment results can be to factors such as anxiety of the examinee and inconsistency of the examiners that need re-evaluation [33, 34].

### Validity

The validity of structured viva in the reviewed health professions research was estimated by the correlation coefficient i.e. the linear relation to a criterion variable. There was a highly significant correlation coefficient (r = 0.52, p < 0.01) between the results of structured viva and multiple-choice questions (MCQs) [18].

Correlation means linear relationship and positive correlation means the linear relationship is in a positive way and that indicates a degree of similarity. MCQs are considered as a standard assessment tool in validity and reliability in health professions education. A positive correlation with high significance in p-value means that structured viva is positively related to one of the highest validity tools (MCQs) – a criterion variable – in health professions education.

Another study stated a positively statistically significant correlation of (0.48 to 0.51) with MCQs and Objective structured clinical examination (OSCE) [19]. Thus, it is another positive correlation between MCQs and one of the best clinical assessment tools (OSCE) in terms of standard validity and reliability.

Both studies above described the validity of structured viva by the correlation coefficient i.e. statistical linear relation with standard assessment tools like MCQs and OSCE, the correlation coefficient was positive with a highly significant p-value. This positive correlation with standard assessment tools may be considered as a statistical parameter of acceptable and good validity of structured viva.

A positive highly significant correlation (r = 0.442, P = 0.001) was seen between marks scored in structured viva and structured theory exam while it was not significant (r = 0.202, P = 0.151) for the marks of traditional viva and structured theory exam, this indicates how far traditional viva is low compared to structured viva in terms of validity as statistical correlation [20].

These numbers in the results may help in the resolution of the conflicts and debates in medical education in different studies regarding the validity and reliability of the traditional oral examination and the structured oral examination for example in the study of de Silva V et al. which defended the traditional oral examination in psychiatric post-graduate exam but also stated to find ways to improve reliability and validity [33]. The ways to improve the traditional oral examination can be - as researchers in this study suggest - the conversion of a traditional oral examination to a structured oral examination.

### Reliability

The reliability of the assessment tool is the ratio of the true score variance to the observed score variance. Reliability types include; internal consistency reliability, inter-rater reliability, and inter-case reliability.

Reliability in terms of internal consistency is measured by Cronbach alpha (Cronbach alpha 0.7 or greater is acceptable, 0.8 or greater is good and 0.9 or greater is excellent). The reliability of structured viva in the reviewed health professions researches was determined by Cronbach alpha and the correlation coefficient.

Cronbach alpha of structured viva was α = 0.80 compared to α = 0.50 to the traditional viva as described by Madhukumar et al. in 2022, while it was α = 0.75 as described by Anastaki et al. [1, 19]. It reached as high as α = 0.79 for parts of Jefferies et al. 2011 exam of postgraduate training [21]. Cronbach alpha is considered high when it is (0.8 or more), all reviewed studies had relatively high values of Cronbach alpha which indicates good reliability of structured viva as an assessment tool in health professions education.

The reliability coefficient may be used to measure the reliability [5]. In the study by Tutton et al. reliability coefficient was (0.7 to 0.8) for the use of a structured rating procedure for viva compared to (0.3 to 0.4) for unstructured viva exam while the multiple-choice test questions (MCQs) is usually (> 0.8) [5]. These high values of reliability coefficient compared with low values for unstructured or traditional viva indicates the superiority of structured viva over traditional viva in terms of reliability.

A study showed significant Inter-rater reliability for each pair of examiners (r = 0.78 to 0.91; p < 0.0001) [16]. One study by Hye Rin Roh et al. stated the inter-rater agreement percentage was 86.7% in their structured viva exam [22]. Another study showed Correlations of > 0.4 in 80% of the scores and > 0.7 in 50% indicating fair to good intra-rater and inter-rater reliability using the structured oral format [34].

Overall judgment after review of the studies is that structured viva has acceptable and good reliability when compared to traditional viva which has low reliability.

### Acceptability

Health professions education is in the era of “Constructivism” and “adult learning theories” where the learners are; self-motivated, problem-based, aware, and have the rights to be involved in the curriculum delivery and assessment tools [1]. This important cornerstone in modern health professions education raised the term “acceptability”.

It is vital for the educational procedure that the learners should be involved and highly accept learning methods and assessment tools. Learners’ acceptability to the new assessment tool, for example, structured viva compared to the traditional or conventional viva vary from a high acceptance rate to a low acceptance rate throughout the published studies, thus it was important to calculate one overall acceptance percentage.

Overall acceptability or the learners’ perception – that the assessment tool is clear, fair, reasonable level of difficulty, acceptance to introduce it in the curriculum or overall acceptance - of structured viva was calculated using the forest plot of 12 researches numeric data in OpenMeta [Analyst] app version Windows 10 and it was (79.8%, P < 0.001) using binary random-effects model due to the heterogeneity of the data (I^2 = 93.506, P < 0.001). This around 80% acceptability rate is considered high and reasonable for an assessment tool i.e. the majority of the participants or the examinees accept structured viva and commented positively regarding it.

Finally, after these results in addition to the traditional viva value of testing higher cognitive levels, problem-solving and communication skills: structured relatively have high acceptability, good validity, and reliability. This outcome makes structured viva a successful alternative preserving traditional viva pros of testing higher cognitive levels and adding to it; validity, reliability, and acceptability thus eliminating its cons.

### Limitations

There were few studies that systematically calculated structured viva statistical parameters of the validity and reliability; thus, the meta-analysis effect size could not be calculated for the validity and reliability. Also, there was limited access to some of the published studies.

## Conclusion

This review showed that structured viva has acceptable validity and reliability as an assessment tool in health professions education compared to traditional viva. There was high learners’ acceptability of structured viva among learners in health professions education who participated as examinees in structured viva. The researchers recommend converting all traditional viva to structured viva/oral exams to be fair and to avoid subjectivity, low validity, and reliability. More researches should be done to calculate the overall statistical effect size for validity and reliability.

## Data Availability

The datasets used during the current study are available from the corresponding author upon reasonable request.

## Abbreviations

BEME: Best Evidence Medical Education
PRISMA: Preferred Reporting Items for Systematic Reviews and Meta-analysis
MCQs: Multiple Choice Questions
BC: Before Christ
OSCE: Objective Structured Clinical Examination
OSPE: Objective Structured Practical Examination
MERSQI: Medical Education Research Study Quality Instrument
